# Gut Microbiota, Short-Chain Fatty Acids, and Herbal Medicines

**DOI:** 10.3389/fphar.2018.01354

**Published:** 2018-11-23

**Authors:** Wuwen Feng, Hui Ao, Cheng Peng

**Affiliations:** ^1^School of Pharmacy, Chengdu University of Traditional Chinese Medicine, Chengdu, China; ^2^Innovative Institute of Chinese Medicine and Pharmacy, Chengdu University of Traditional Chinese Medicine, Chengdu, China; ^3^State Key Laboratory Breeding Base of Systematic Research, Development and Utilization of Chinese Medicine Resources, Chengdu University of Traditional Chinese Medicine, Chengdu, China

**Keywords:** gut microbiota, short-chain fatty acids, herbal medicines, traditional Chinese medicine, metabolites

## Abstract

As an important source for traditional medical systems such as Ayurvedic medicine and traditional Chinese medicine, herbal medicines have received widespread attentions from all over the world, especially in developing countries. Over the past decade, studies on gut microbiota have generated rich information for understanding how gut microbiota shape the functioning of our body system. In view of the importance of gut microbiota, the researchers engaged in studying herbal medicines have paid more and more attention to gut microbiota and gut microbiota metabolites. Among a variety of gut microbiota metabolites, short-chain fatty acids (SCFAs) have received most attention because of their important role in maintaining the hemostasis of hosts and recovery of diseases. Herbal medicines, as an important resource provider for production of SCFAs, have been demonstrated to be able to modulate gut microbiota composition and regulate SCFAs production. In this mini-review, we summarize current knowledge about SCFAs origination, the role of SCFAs in health and disease, the influence of herbal medicine on SCFAs production and the corresponding mechanisms. At the end of this review, the strategies and suggestions for further research of SCFAs and herbal medicines are also discussed.

## Introduction

Herbal medicines, also named botanical medicines or phytomedicines, are one kind of medicines that are derived from plants. Herbal medicines can be purified herbal compounds, crude herbal extractions, herbal formulas, etc. In some cases, the concept of herbal medicines can be extended to medicinal materials of fungal, mineral, and animal origin (World Health Organization, 2000). Herbal medicines have been extensively used in traditional medical systems such as Ayurvedic medicine, traditional Chinese medicine, and Unani medicine, for over hundreds of or even thousands of years. It is estimated that about 75–80% of the world population rely on herbal medicines for primary health care (Kamboj, 2000). In drug discovering area, compounds isolated from herbal medicines have been studied as lead compounds for drug discovery. For example, about 63% anticancer small molecules approved by the US Food and Drug Administration are derived from herbal medicines (Boufridi and Quinn, 2018). Although the efficacy of herbal medicines in fighting against diseases has been established, the mechanisms of how they exert their therapeutic effects remain unclear.

In recent years, gut microbiota has emerged as an important frontier in understanding the homeostasis of human body and the development of diseases. Studies in recent years have showed that gut microbiota can metabolize various food and medicinal molecules and generate a series of metabolites such as short-chain fatty acids (SCFAs), indole derivatives, and polyamines, to regulate the homeostasis of human body and progress of diseases (Nicholson et al., 2012; Postler and Ghosh, 2017). Among those gut microbiota metabolites, SCFAs have received widespread attention because of their significant physiological and pharmacological effects. Since SCFAs might be linked to the therapeutic effects of herbal medicines, a growing number of studies have investigated the effect of herbal medicines on gut microbiota composition and SCFAs production in recent years. In this review, we focus on the progress of studies concerning biological effects of SCFAs and the effects of herbal medicines on gut microbiota composition and SCFAs production. Additionally, the suggestions for the future research of herbal medicines and SCFAs are also provided in this paper.

## Gut microbiota and metabolites

Gut microbiota, also named as intestinal microbiota, intestinal flora, or gut flora, is a large and diverse group of microorganisms that live in the gastrointestinal tract. The human body contains about 10^14^ microorganisms in the intestine, which is 10 times greater than the total number of human cells in the body. It contains taxa that include bacteria, eukaryotes, viruses, and even archaeon with over 35,000 bacterial species in collective human bodies (Sekirov et al., 2010). Bacteria is the major constituent part of microbiota in the colon, where around 300–500 different species live. It is reported that there are over 50 bacterial phyla, yet the human gut microbiota mainly contains two bacterial phyla, *Firmicutes* and *Bacteroidetes*, and 99% of the bacteria come from about 30 or 40 species (Lozupone et al., 2012). A large number of studies have reported that food and medicines have significant influences on gut microbiota, including the composition and metabolism of gut microbiota. Meanwhile, the human body and gut microbiota co-produce a series of metabolites that act as shuttling information between human cells and gut microbiota, thus, a large portion of beneficial or detrimental effects of food and medicines on human body could be achieved (Etxeberria et al., 2013).

In light of the importance of metabolites produced by gut microbiota, numerous studies have been conducted to identify those metabolites over the past two decades. Those metabolites mainly include SCFAs, indole derivatives, polyamines, organic acids, vitamins (Table 1). According to classification method put forward by Postler and Ghosh (2017), we divide those metabolites into three types with some minor modification: (I) metabolites that are transformed by gut microbiota from drugs or dietary components; (II) metabolites that are secreted by host and modified by gut microbiota; (III) metabolites that are synthesized by gut microbiota *de novo*. An example for the first type of metabolites is compound K, which is transformed by gut microbiota from ginsenosides Rb1, Rb2, and Rc (Kim et al., 2013). The typical examples for the second type of metabolites are secondary bile acids that are synthesized from cholesterol in the liver and transformed by gut microbiota (Sayin et al., 2013), and the examples for the third type of metabolites include SCFAs and polysaccharide A.

**Table 1:** Metabolites of gut microbiota.

**Metabolites**	**Molecular targets**	**Producers**	**Effects on hosts**	**Key references**
Short-chain fatty acids (acetate, propionate, butyrate, iso-butyrate, valerate, iso-valerate, hexanoate)	GPR43/FFAR2, GPR41/ FFAR3, GPCR109A/HCA2, GPCR81/HCA1, HDAC1, and HDAC3	*Bacteroidetes*: acetate and propionate; *Firmicutes*: butyrate	Decrease colonic pH; inhibit growth of pathogens; improve integrity and function of colonic epithelial cells; anti-lipolysis; increase insulin sensitivity and energy expenditure; inhibit production of proinflammatory cytokines. Involved in diabetes, ulcerative colitis, radiation proctitis, Crohn's disease, colorectal cancer, Parkinson's disease, asthma.	Kendrick et al., 2010; Tan et al., 2014; Bolognini et al., 2016; Morrison and Preston, 2016; Vernocchi et al., 2016; Huang et al., 2017; van der Beek et al., 2017
Indole derivatives (indole, indoleacetylglycine, indoxyl sulfate, indole-3-propionate, 6-sulfate, serotonin)	Aryl hydrocarbon receptor, nuclear receptor subfamily 1 group I member 2	*Clostridium sporogenes, Escherichia coli*	Enhance colon integrity; modulate expression of proinflammatory and anti-inflammatory genes; implicated in brain-gut axis.	Keszthelyi et al., 2009; Kiss et al., 2011; Venkatesh et al., 2014
Biogenic amines (trimethylamine-N-oxide (TMAO), trimethylamine, agmatine, cadaverine, putrescine, spermidine, spermine, histamine)	Histamine receptors	*Campylobacter jejuni, Clostridium saccharolyticum, Faecalibacterium prausnitzii, Bifidobacterium*	Involve in intestinal epithelial integrity, cell-growing, and aging; modulate anti-inflammatory and antitumoral effects. TMAO is positively correlated with atherosclerosis, non-alcoholic fatty liver disease, diabetes, and impaired renal function.	Hanfrey et al., 2011; Levy et al., 2016; Ost and Round, 2018
Secondary bile acids (taurocholate, cholate, deoxycholate, chenodeoxycholate, α-muricholate, β-muricholate, glycocholate, taurochenoxycholate, glycochenodeoxycholate, taurocholate, tauro–α-muricholate, ursodeoxycholate, hyodeoxycholate, glycodeoxylcholate, taurohyocholate)	G protein-coupled bile acid receptor 1, bile-acid-synthesis controlling nuclear receptor farnesoid X receptor	*Lactobacillus, Bifidobacteria, Enterobacter, Bacteroides, Clostridium, Eubacterium*, and *Escherichia*,	Regulation of cholesterol, glucose, and energy homeostasis; facilitate lipid and lipid-soluble vitamins assimilation; maintain intestinal barrier function; inhibit NF-*k*B-dependent transcription of proinflammatory genes.	Ridlon et al., 2006; Dawson et al., 2009; Sayin et al., 2013; Guo C. et al., 2015
Vitamins (vitamin B9, thiamine, vitamin B2, biotin, vitamin B12, niacin, pyridoxine, vitamin K, vitamin B1, vitamin B5, vitamin B8, folate, riboflavin)	Vitamin receptors	*Bifidobacterium bifidum, Bifidobacterium breve, Bifidobacterium adolescentis, Bacillus Subtilis, Escherichia coli, Bacteroidetes, Fusobacteria, Proteobacteria*, and *Actinobacteria*	Implicated in cellular metabolism; strengthen immune function; regulate cell proliferation; provide vitamin sources for hosts.	Deguchi et al., 1985; Noda et al., 1994; Pompei et al., 2007; Said, 2011; Magnúsdóttir et al., 2015
Lipids (LPS, glycerol, acylglycerols, sphingomyelin, cholesterol, triglycerides)	CD14/Toll-like receptor 4 (LPS), Niemann–Pick C1-like cholesterol receptor (cholesterol)	*Roseburia, Lactobacillus, Bifidobacterium, Klebsiella, Enterobacter, Citrobacter*, and *Clostridium*	LPS can promote inflammatory response and is involved in insulin resistance, obesity, type 2 diabetes mellitus, alcoholic liver disease, non-alcoholic fatty liver disease, chronic hepatitis C.	Cani et al., 2007; Holmes et al., 2011
Polyphenol	-	*Escherichia coli, Salmonella, Pseudomonas, Clostridium, Bacteroides*	Regulate gut microbiota composition and activity; possess antioxidant activity; reduce colon cancer risk; decrease inflammatory factors.	Etxeberria et al., 2013

For herbal compounds, they can be transformed by gut microbiota (type I metabolites) or be metabolized to other types of metabolites of new backbones (type III metabolites). The reactions that transform the herbal compounds include glycoside hydrolase, oxidation, reduction, isomerization, ester hydrolysis, rearrangement, esterification, intramolecular cyclization, and condensation (Xu et al., 2017). Those transformed metabolites might exhibit different bioactivity and bioavailability compared with their precursors. For example, gut microbiota could hydrolyze glycyrrhizin to 18β-glycyrrhetic acid, a compound that can be absorbed more easily compared with glycyrrhizin (Akao et al., 1994). The metabolites that are transformed from herbal medicines have been partly reviewed (Wang et al., 2011; Chen et al., 2016; Xu et al., 2017). SCFAs are the mainly *de novo* synthesized metabolites that are associated with herbal medicines. Among those two types of metabolites, SCFAs have caught much attention over the past decade in herbal research area.

## Production of SCFAs

SCFAs are one class of the most-thoroughly studied gut microbiota metabolites. They are saturated aliphatic organic acids with a backbone of one to six carbons (Cait et al., 2017). SCFAs are primarily produced in large intestine by gut microbiota fermentation of plant-derived carbohydrates that have escaped digestion and absorption in small intestine although non-digested proteins or peptides could also contribute to production of SCFAs (Blachier et al., 2007). Dietary proteins and peptides that contain branched-chain amino acids can be metabolized to SCFA, but they are typically metabolized as branched-chain fatty acids such as 2-methylbutyrate, iso-butyrate, and iso-valerate. Human body cannot produce enzymes that can catalyze fermentation of carbohydrates while gut microbiota can secrete a serious of enzymes such as propionate-CoA transferase and propionaldehyde dehydratase to metabolize carbohydrates into SCFAs (Flint et al., 2014; Fernández et al., 2016).

Acetate, propionate, and butyrate are the most abundant SCFAs in gastrointestinal tract (≥95%), whereas formate, valerate, caproate, etc., make up the remaining (den Besten et al., 2013). Acetate and propionate are mainly produced by *Bacteroidetes* whereas *Firmicutes* are the primary contributors of butyrate (Levy et al., 2016). Studies have found out that acetate is produced by most of the enteric bacteria such as *Lactobacillus* spp., *Bifidobacterium* spp., *Akkermansia muciniphila, Bacteroides* spp., *Prevotella* spp., *Ruminococcus* spp., and *Streptococcus* spp. via Wood-Ljungdahl pathway and acetyl-CoA pathwy (Louis et al., 2014; Fernández et al., 2016). Propionate is produced by *Phascolarctobacterium succinatutens, Bacteroides* spp., *Dialister* spp., *Megasphaera elsdenii, Veillonella* spp. *Coprococcus catus, Roseburia inulinivorans, Ruminococcus obeum, Salmonella* spp., via succinate pathway, acrylate pathway, and propanediol pathway (Koh et al., 2016). Butyrate is produced by *Roseburia* spp. *Eubacterium rectale, Clostridium leptum, Eubacterium hallii, Coprococcus eutactus, Faecalibacterium prausnitzii, Eubacterium rectale, Anaerostipes caccae*, and *Coprococcus catus* via the butyryl-CoA:acetate CoA-transferase routes and the phosphotransbutyrylase/butyrate kinase routes (Fernández et al., 2016; Koh et al., 2016). The molar ratio of acetate, propionate, and butyrate in the colon and stool is about 3:1:1 (Canfora et al., 2015). The molar ratio and content of SCFAs released in large intestine is influenced by several factors that include but not limited to the strain and quantity of gut microbiota, substrate source, host genotype, and intestinal transit time (Wong et al., 2006).

## SCFAs in health and diseases

In gastrointestinal tract, SCFAs can reduce the luminal pH and enhance the absorption of some nutrients (Macfarlane and Macfarlane, 2012). In addition, SCFAs can directly impact gut microbiota composition. On one hand, SCFAs could serve as a carbon source for gut microbiota, on the other hand, SCFAs at high concentration exhibit toxic effects on some gut microbiota species (Sun and O'Riordan, 2013). Specifically, non-ionized SCFAs in gastrointestinal tract could diffuse across bacterial membrane and lead to a series of effects ranging from alteration of osmotic balance, DNA synthesis, amino acid uptake, and oxidative metabolism to chemotactic responses (Sun and O'Riordan, 2013). For example, at higher concentration and low pH condition, SCFAs could strongly inhibit the growth of *Salmonella*, a common foodborne pathogen, via reducing the expression of invasion genes (El-Gedaily et al., 1997).

Cecum and large intestine can efficiently and rapidly absorb the generated SCFAs by colonocytes, with only about 5% SCFAs secreted in the feces (Topping and Clifton, 2001). A small portion of SCFAs that exist in unionized form can directly cross epithelial barrier via non-ionic diffusion, whereas most SCFAs exist in ion state and their entrance into hosts usually needs specialized transporters. Those transporters include hydrogen-coupled monocarboxylate transporter 1 (MCT 1), MCT 2, MCT 4, and sodium-coupled monocarboxylate transporter 1 (Ritzhaupt et al., 1998; Moschen et al., 2012). Of the three major SCFAs, butyrate is the preferred material for colonocytes as an important energy source, and it is largely metabolized in epithelial mucosa in maintenance of colonic health (van der Beek et al., 2017). On the contrary, propionate and acetate can travel across the epithelium to the liver, where most of propionate is metabolized but not acetate. As a result, acetate is the most abundant SCFA in the circulation system whereas only a small amount of butyrate and propionate could be found in periphery (Koh et al., 2016).

In large intestine, epithelial cells could secrete antimicrobial peptides which are important for defending against attachment and invasion of external or internal pathogens. This process is associated with induction of LL-37 production (Termén et al., 2008). In addition, SCFAs can maintain the integrity of the epithelial barrier by regulation of tight junction proteins including claudin-1, occludin and Zonula Occludens-1 (Wang et al., 2012). Decrease of those proteins would facilitate translocation of bacteria and lipopolysaccharide (LPS) to trigger inflammatory reaction. Furthermore, SCFAs could increase cell membrane assembly, mucosal cell migration, and proliferation and differentiation of colonocytes. SCFAs could also protect colonic epithelium via enhancement of the expression of mucin 2, modulation of oxidative stress and immune response (van der Beek et al., 2017). These role of SCFAs are especially important for human body to fight against intestinal diseases such as Crohn's disease, ulcerative colitis, and colorectal cancer.

**Figure 1:**
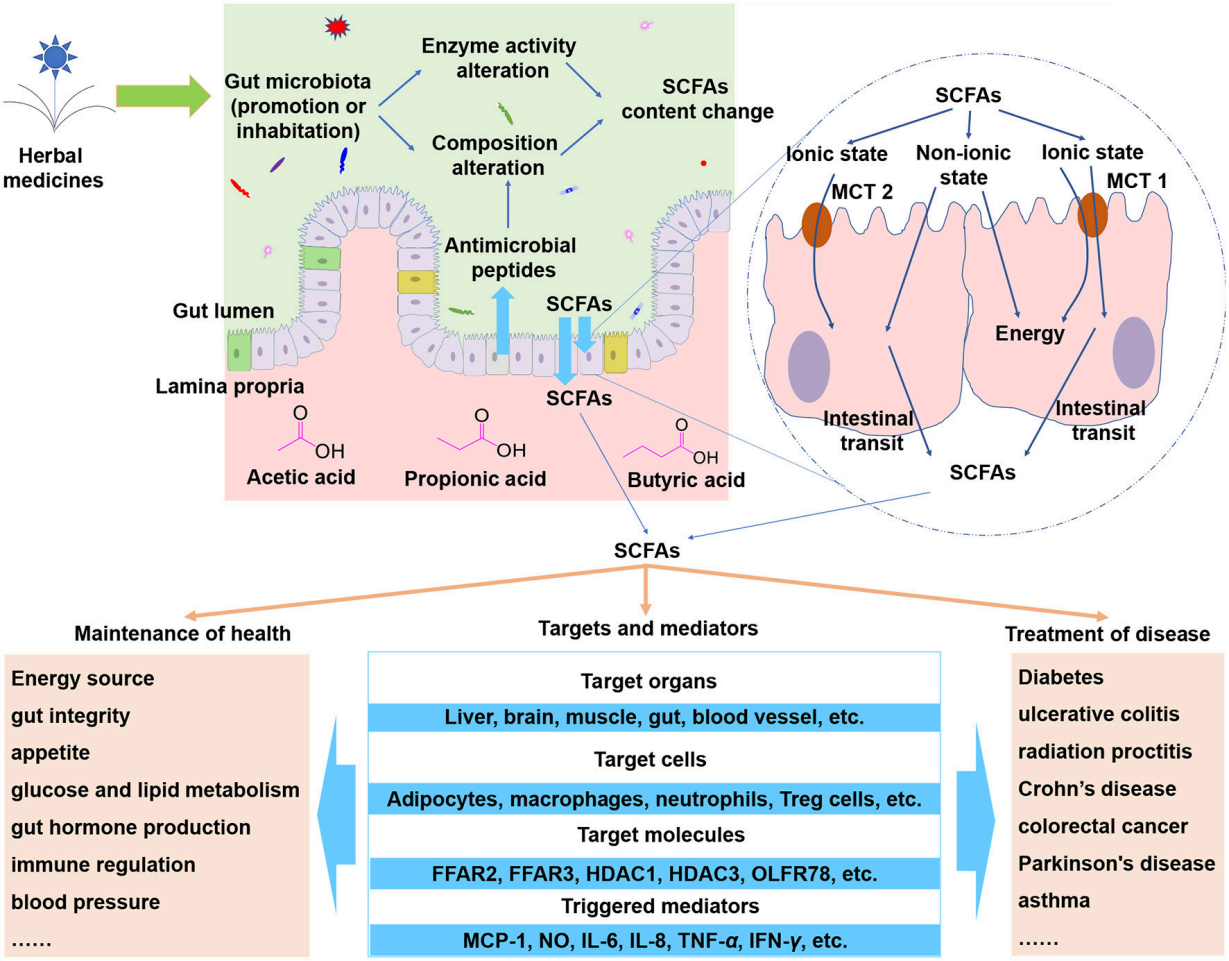
SCFAs are important metabolites of herbal medicines. When herbal compounds enter into gut lumen, the composition and the enzyme activity of gut microbiota could be modulated and thus the SCFAs could be influenced. SCFAs can cross epithelial barrier via non-ionic diffusion or transporters such as hydrogen-coupled monocarboxylate transporter 1 (MCT 1), MCT 2, and MCT 4. The absorbed SCFAs can be transported to target organs and cells to trigger a wide range of effects. Abbreviations: FFAR2, free fatty acid receptors 2; FFAR3, free fatty acid receptors 3; HDAC1, nuclear class I histone deacetylase 1; HDAC3, nuclear class I histone deacetylase 3; OLFR78, olfactory receptor 78; MCP-1, macrophage chemoattractant protein-1; NO, nitric oxide; IL-6, interleukin-6; IL-8, interleukin-8; TNF-α, tumor necrosis factor-α; IFN-γ interferon-γ.

After being absorbed, SCFAs could be transported to different organs. Propionate will mainly participate in gluconeogenesis while acetate and butyrate will mainly participate in lipid biosynthesis. Apart from directly serving as important energy resources, SCFAs could modulate various biological response of hosts that include inflammation, oxidative stress, and so on (Tan et al., 2014; Huang et al., 2017). In general, two different mechanisms are involved in those processes. The first kind of mechanisms is associated with direct activation of a certain G-protein-coupled receptors (GPCRs), and the second mechanisms is associated with direct inhibition of nuclear class I histone deacetylases (HDACs) which happen inside the cells (Tan et al., 2014). Those GPCRs include GPCR109A, free fatty acid receptors 2 (FFAR2, also named as GPCR43), 3 (FFAR3 or GPCR41) which are involved in regulation of glucose and lipid metabolism, inflammation, oxidative stress, and so on (Tan et al., 2014; Huang et al., 2017). The HDACs include HDAC1 and HDAC3 whose inhibition are mainly associated with anti-inflammatory immune phenotype (Tan et al., 2014), including decreasing of proinflammatory cytokine (interleukin-6, interleukin-8, tumor necrosis factor-α, etc.) (Kendrick et al., 2010), reducing NF-*k*B activity (Usami et al., 2008). In healthy state, SCFAs serve as key regulators for modulation of gut integrity, appetite, energy homeostasis, gut hormone production, and immune function (Bolognini et al., 2016; Morrison and Preston, 2016). While in disease state, SCFAs can exert diverse protective effects against diabetes, ulcerative colitis, radiation proctitis, Crohn's disease, colorectal cancer, Parkinson's disease, asthma, and so forth (Vernocchi et al., 2016; van der Beek et al., 2017). The roles and mechanisms of SCFAs on hosts are summarized in Figure 1.

## Herbal medicine can modulate gut microbiota composition

So far, many diseases have been demonstrated to be associated with gut microbiota. Those diseases include ulcerative colitis, cancer, chronic kidney disease, Alzheimer's disease, obesity, diabetes, etc. In recent years, a large number of studies have showed that herbal medicines including single compounds, single herbals and herbal formulas, are capable of reversing the abnormal gut microbiota composition in diseased human cohorts and model animals (Chen et al., 2016). Some studies even use germ-free animal or fecal transplantation to prove the decisive role of gut microbiota in treatment of diseases. For example, Chang et al. used fecal transplantation method and proved that high molecular weight polysaccharides isolated from *Ganoderma lucidum* could reduce obesity in mice via modulating the composition of the gut microbiota (Chang et al., 2015). Kato et al. used germ-free mice and regular mice to study the effect of a multi-herbal medicine Juzentaihoto on heat shock gene expression. They found out that Juzentaihoto could change the gut microbiota, and gut microbiota could, in turn, change the heat shock gene expression (Kato et al., 2007).

Most of the herbal medicines are taken orally. After oral administration, the components of herbal medicines can reach colon where most gut microbiota dwell, especially for the herbal compounds that are difficult to absorbed by human body. For example, it is estimated that only 5–10% of polyphenols (such as curcumin, quercetin, and trans-resveratrol) intake can be absorbed in small intestine (Cardona et al., 2013). Berberine, an herbal compound that possesses strong cholesterol-lowering effects in clinic, only shows < 1% oral bioavailability (Chen et al., 2011). In fact, most herbal medicines exhibit poor oral bioavailability (Chen et al., 2016). These molecules in herbal medicines could easily pass small intestine and accumulate in colon, a cozy environment for direct contact and dual interaction between unabsorbed compounds and gut microbiota. As a result, the gut microbiota composition could be modulated.

Although some new species could be colonized in gastrointestinal tract and some species could be completely eliminated after intake of herbal medicines, the modulating mechanisms of herbal medicines on gut microbiota mainly include two types: direct or indirect promote or inhibit the growth of specific species (Xu et al., 2017). For example, *Red Ginseng* (the streamed root from *panax ginseng* C.A. Mey.) and *Semen Coicis* [dried seed of *Coix lacryma-jobi* L. var. *mayuen* (Roman.) Stapf] could promote the growth of probiotics (microorganisms that are of beneficial effects to hosts) *Bifidobacterium* and *Lactobacillus* while could inhibit the growth of some pathogen strains *in vitro* (Guo M. et al., 2015). In fact, a relative large number of herbal medicines have been demonstrated to be able to promote probiotics and inhibit pathogens (Chen et al., 2012). The ability of herbal medicines to directly inhibit gut microbiota species can be easily understood as many herbal compounds such as essential oils possess microorganism killing activities (Burt, 2004). Herbal medicines can also directly promote the growth of some gut microbiota species by acting as prebiotics (Singdevsachan et al., 2016). The indirect promotive and inhibitive role of herbal medicines on gut microbiota includes preventing the colonization of extraneous or indigenous pathogen via competitive exclusion, competitive consumption of nutrients, and induction of host immune response (Kamada et al., 2013). By modulation of gut microbiota structure, the metabolites of gut microbiota that are beneficial or detrimental to hosts can also be influenced. Those beneficial metabolites can (partly) attribute to the therapeutic effects of herbal medicines.

Worth notice is that although many studies reported the beneficial effects of herbal medicines on gut microbiota, herbal medicines can also induce negative effects on gut microbiota. *Indigo naturalis* (Qing dai) is an ancient herbal medicine that has been used as an anti-inflammatory agent to treat skin rash, dermatitis, upper airway inflammation etc. Adachi et al. found out that *Indigo naturalis* could significantly aggravate colitis induced by oxazolone, and this process is associated with modulation of gut microbiota (Adachi et al., 2017). *Ganoderma lucidum* is traditionally used for promoting health and longevity, but a recent study found out that *Ganoderma lucidum* also has detrimental effects. It can increase the opportunistic pathogens including *Acinetobacter, Pseudomonas, Stenotrophomonas, Peptococcus*, and *Serratia* (Wu et al., 2017).

## Herbal medicines can modulate SCFAs production

Just as the food does, herbal medicines also contain many carbohydrates that can be utilized for production of SCFAs. For herbal medicines, they contain two distinctive compounds that can be metabolized into SCFAs, one is the glycosides which contain sugar moieties that can be metabolized by gut microbiota (Xu et al., 2017), the other is carbohydrates such as pectin, resin and fiber. Both carbohydrates and glycosides ubiquitously exist in herbs. Except for a few herbal medicines that only contain one or a few of specific compound(s), most herbal medicines are made from total extracts of crude herbs. Therefore, herbal medicines can naturally act as a resource provider for gut microbiota to produce SCFAs considering that most orally taken herbal medicines contain carbohydrates and glycosides.

In addition to acting as a resource provider for generation of SCFAs, herbal medicine can also intervene the generation of SCFAs by modulation of the composition of gut microbiota and the bioactivity of enzymes which catalyze the production of SCFAs. In gastrointestinal tract, a lot of gut microbiota species and enzymes are responsible for generation of SCFAs, and the degradation process of carbohydrates is species specific and enzyme specific (Kaoutari et al., 2013; Tan et al., 2014). Here we listed the studies that have investigated the influence of herbal medicines on both the composition of gut microbiota and production of SCFAs (Table 2). Some other studies also investigated the effect of herbal medicines on production of SCFAs but not gut microbiota (Hu et al., 2012; Tong et al., 2016). The increase of SCFAs might be linked to the increase of both the bioactivity or/and concentration of enzymes and the number of microorganisms responsible for generation of SCFAs. Although there is a lack of evidence for increased enzyme activity after herbal medicine treatment, the influence of the enzyme on SCFAs production has been established (Andersch et al., 1983). Modulation of gut microbiota to influence the generation of SCFAs is especially important for herbal medicines that contain molecules without sugar moieties. For example, berberine is an alkaloid compound with anti-obesity ability and SCFAs increasing ability. Although its molecular structure doesn't contain sugar moiety, it can selectively increase SCFA-producing bacteria *Blautia* and *Allobaculum* (Zhang et al., 2012).

**Table 2:** The effect of herbal medicines on gut microbiota and short-chain fatty acids (SCFAs).

**Herbal medicines (compounds)**	**Model**	**Typical effect on gut microbiota**	**Effect on fecal SCFAs**	**References**
Berberine	High-fat diet-fed male Wistar rats	SCFAs producing bacteria *Allobaculum, Blautia, Bacteroides*, etc. were enriched.	Increased total SCFAs, mainly acetic acid and propionic acid	Zhang et al., 2012
Xiexin Tang (Rhei rhizome, Scutellaria radix, Coptidis rhizome)	High-fat diet-induced type 2 diabetic male Sprague-Dawley rats	Increased *Proteobacteria* and *Actinobacteria* at the phylum level; increased *Alloprevotella, Barnesiella*, [Eubacterium] *Ventriosum* group, *Lachnospiraceae* UCG-001, *Papillibacter* and *Prevotellaceae* NK3B31 group at genus level	Increased acetic acid, propionic acid, isobutyric acid, butyric acid	Wei et al., 2018
Polysaccharides from *Chrysanthemum morifolium*	Dextran sulfate sodium induced male C57BL/6 colitis mice and male Sprague-Dawley colitis rats	Increased *Butyricicoccus, Clostridium, Lactobacillus, Bifidobacterium, Lachnospiraceae* and *Rikenellaceae*; decreased *Escherichia, Enterococcus*, and *Prevotella*	Increased acetic acid, propionic acid, butyric acid, isobutyric acid, valeric acid, and isovaleric acid	Tao et al., 2016, 2017
Total saponins from *Polygonatum kingianum*	High-fat diet-induced type 2 diabetic male Sprague-Dawley rats	Low and high dose decreased *Bacteroidetes, Proteobacteria* and increased *Firmicutes*; Low dose increased *Ruminococcaceae* family and *Ruminococcus* genus; high dose increased *Veillonellaceae* family and *Anaerovibrio* genus	Low dose decreased total SCFAs, acetate acid, propionate acid, and butyrate acid; high dose increased total SCFAs and propionate acid	Yan et al., 2017
Polysaccharides from *Polygonatum kingianum*	High-fat diet-induced type 2 diabetic male Sprague-Dawley rats	Decreased *Bacteroidetes, Proteobacteria* and increased *Firmicutes*; increased *Ruminococcaceae* family and *Ruminococcus* genus	Decreased total SCFAs, acetate acid, propionate acid, and butyrate acid	Yan et al., 2017
Homogeneous polysaccharide, S-3-1, from Sijunzi Decoction	Artificial gastric juice, intestinal juice, and human fecal microflora	S-3-1 could modulate *Lactobacillus, Pediococcus, Streptococcus, Bacteroides, Enterococcus, Clostridium, Dorea, Paraprevotella* and *Oscillospira*; incubated S-3-1 could regulate the abundance of *Lactobacillus, Pediococcus, Streptococcus*, etc.	S-3-1 showed no influence on total SCFAs; incubated S-3-1 elevated acetic acid and total SCFAs	Gao et al., 2018
Polysaccharides S-3 from Sijunzi decoction	Reserpine-induced spleen deficiency Wistar rat	Restored the disturbance of gut microbiota induced by reserpine	Increased acetic acid, butyric acid and propionic acid	Wang et al., 2016
Chaihu-Shugan-San Decoction (Bupleuri Radix, Chuanxiong Rhizoma, etc. six herbals)	High-fat diet-induced non-alcoholic fatty liver disease male Sprague-Dawley rats	Decreased *Enterobacteriaceae, Staphylococcaceae* families and *Veillonella* genus; increased *Anaeroplasma* genus	A trend to increase butyric acid, but no difference was observed	Liang et al., 2018
Hydroxysafflor yellow A	High-fat diet-induced obese C57BL/6J mice	Increased genera *Akkermansia, Romboutsia, Butyricimonas* and *Alloprevotella*; decreased the phyla *Firmicutes*/*Bacteroidetes* ratio	Increased acetic acid, propionic acid, and butyric acid	Liu et al., 2018
Leaves of *Passiflora edulis*	Normal male Wistar rats	Increased *Bifidobacterium, Lactobacillus*, total aerobic bacteria and *Enterobacteriaceae*	Decreased acetic acid and butyric acid	da Silva et al., 2013
Polysaccharides (H1) isolated from *Hirsutella sinensis*	High-fat diet-induced obese C57BL/6J male mice	Promoted the growth of *Parabacteroides goldsteinii*	No obvious effect on SCFAs	Wu et al., 2018
Polysaccharides (CYP3) from Chinese Yam	Normal weanling Sprague-Dawley rats	Increased beneficial gut microbiota, but suppressed bacterial pathogens	Increased acetate and butyrate	Kong et al., 2009
Polysaccharide peptide form *Trametes versicolor* Extract	Fresh fecal samples from healthy volunteers	Increased *Bifidobacterium* spp. and *Lactobacillus spp*.; reduced *Clostridium* spp., *Staphylococcus* spp. and *Enterococcus* spp.	Increased acetate, propionate, butyrate	Yu et al., 2013
Lotus Seed Resistant Starch	Normal male BALB/c mice	Increased *Lactobacillus, Bifidobacterium, Lachnospiraceae, Ruminococcaceae*, and *Clostridium*; decreased *Rikenellaceae* and *Porphyromonadaceae*	Increased formic, acetic, propionic, butyric, isobutyric, and lactic acids	Zeng et al., 2017
Xylooligosaccharides from corn cobs	Normal Kunming male mice and *Lactobacillus plantarum*	Increased the viable *Lactobacilli* and *Bifidobacterial*; decreased the viable *Enterococcus, Enterobacter*, and *Clostridia* spp.	Acetate was major metabolites of xylooligosaccharides	Yu et al., 2015
*Coptis chinensis* decoction	Male normal Sprague-Dawley rats	Increased *Acidovorax, Enterobacter* and *Veillonella*; decreased *Bacteroides* and *Prevotella*	Decreased SCFAs	Li et al., 2015
Phlorizin	type 2 diabetic db/db mice and db/+ mice	Increased the gut microbial diversity, increased the growth of *Akkermansia muciniphila* and *Prevotella*	Increased total SCFAs, acetic acid, propionic acid, butyric acid, valeric acid	Mei et al., 2016
Reishi mushroom (*Ganoderma lingzhi*)	High-fat diet supplemented Sprague-Dawley rats	Reduced *Clostridium coccoides* and *Clostridium leptum*; increased *Akkermansia muciniphila*, and *Enterobacteriaceae*	Increased total SCFAs, propionate and acetate	Yang et al., 2017
Carboxymethyl pachyman (a modified polysaccharide from *Poria cocos*)	5-fluorouracil U-treated CT26 tumor-bearing male Balb/c mice	Increased *Bacteroides, Lactobacillus*, butyric acid-producing bacteria and acetic acid-producing bacteri*a*; decreased *Firmicutes* and *Proteobacteria*	Increased acetic acid, propionic acid, and butyric acid; reduced isobutyric acid and isovaleric acid	Wang et al., 2018
Triterpenoids from *Ganoderma lucidum*	High-fat diet fed male Wistar rats	Increased *Alistipes, Peptococcaceae, Defluviitalea* and *Alloprevotella*; decreased *Phascolarctobacterium* and *Clostridium* XVIII	Increased total SCFAs, propionic acid, and butyric acid	Guo et al., 2018
Polysaccharide from *Plantago asiatica* L	High-fat diet-induced type 2 diabetic male Wistar rats	Increased *Bacteroides vulgatus, Lactobacillus fermentum, Prevotella loescheii* and *Bacteroides vulgates*	Increased total SCFAs, propionic acid and butyric acid	Nie et al., 2017
Daikenchuto (TU-100)	Normal male and female C57Bl6/J mice	Increased *Lactococcus* spp., *Clostridium clostridioforme, Clostridium populeti, Roseburia intestinalis, Eubacterium hallii, Clostridium thermocellum*. etc.	Increased total SCFAs, acetate, propionate, butyrate	Hasebe et al., 2016

Most studies reported the beneficial effects of herbal medicines on production of SCFAs, however, herbal medicines can also induce negative effects on SCFAs production. For example, polysaccharides from *Polygonatum kingianum* could decrease total SCFAs, acetate acid, propionate acid, and butyrate acid in high-fat diet-induced type 2 diabetic rats (Yan et al., 2017). Leaves of *Passiflora edulis* could decrease acetic acid and butyric acid in normal male Wistar rats (da Silva et al., 2013). In addition, the effects of herbal medicines on SCFAs production seems to be dose dependent. For example, low dose of total saponins from *Polygonatum kingianum* could decrease total SCFAs, acetate acid, propionate acid, and butyrate acid, whereas high dose could increase total SCFAs and propionate acid (Yan et al., 2017).

## SCFAs and the therapeutic effects of herbal medicines, association or causation?

The association or causation relationship between the change of gut microbiota composition and therapeutic effects has been noticed for a long time (Louis et al., 2014), and so does the relationship between gut microbiota metabolites and therapeutic effects. Even though the therapeutic role of SCFAs in some diseases has been established, it still raises the question of whether the modulated gut microbiota and SCFAs production are responsible for the therapeutic effects of herbal medicines or not. Without further validation, we cannot determine the influenced gut microbiota species are caused by direct effect of herbal medicines or indirect effect of hosts that are intervened by herbal medicines, considering that hosts can also shape the structure of gut microbiota, for example, by secretion of antimicrobial peptide (Round and Mazmanian, 2009). In addition, as SCFAs can be generated in both healthy and disease state, it seems that SCFAs can still contribute to recovery of diseases without herbal medicines' intervention although in some cases only high concentration of SCFAs can induce the effects (Schilderink et al., 2013). Furthermore, even though the causal relationship of herbal medicines, SCFAs, and therapeutic effects has been confirmed, to what extent are the gut microbiota and SCFAs responsible for the therapeutic effects of herbal medicines is another question considering that some herbal compounds can be directly absorbed and exert the therapeutic effects. Therefore, to reach a valid conclusion, further steps such as fecal transplant of gut microbiota, gut microbiota culturing, gnotobiotic model, and germ-free animals are needed.

## Comments on future study

Comprehensive characterizing of herbal compounds is necessary. Unlike chemical medicines with specific ingredients, herbal medicines usually contain many different compounds (Qiao et al., 2016; Yue et al., 2017). Therefore, characterizing of compounds in herbal medicines is a prerequisite step to understand the mechanisms of herbal medicines. While characterizing the chemical compounds of herbal medicines, special attention should be paid to polysaccharides. Because most of the detection methods for herbal medicines are reverse-phase liquid chromatography-based techniques, detection of polar compounds such as polysaccharides would be difficult. However, polysaccharides play important roles in whole therapeutic effects of herbal medicines. For example, ginseng polysaccharides could improve the absorption of ginsenosides, which are bioactive compounds of ginseng *in vivo* (Zhou et al., 2016; Shen et al., 2018). In addition, polysaccharides with different molecular weight can exhibit different bioactivity (Chang et al., 2015; Wu et al., 2018). Therefore, special attention should be paid to compound characterization especially polysaccharides. Furthermore, as aforementioned, polysaccharides could decrease SCFAs even though many studies reported the SCFAs-elevating effects of polysaccharides. And correspondingly, careful attention should be paid to SCFAs detection while studying the herbal medicines containing polysaccharides.

SCFAs, transformed herbal compounds, and prototype compounds, which of them are mainly responsible for therapeutic effects? In general, at least three different types of *in vivo* bioactive compounds are potentially associated with herbal medicines: SCFAs, gut microbiota transformed herbal compounds (such as deglycosylated and isomerized compounds), and untransformed prototype compounds. Further studies are needed to determine which of them is mainly responsible for the curative effects. For example, berberine could be converted into dihydroberberine, berberrubine, demethyleneberberine (Spinozzi et al., 2014; Wang K. et al., 2017). Berberine could also promote SCFAs production (Wang L. L. et al., 2017). These transformed compounds, SCFAs, and prototype berberine can be detected *in vivo*. Since three of them might be responsible for therapeutic effects, determination of which of them is the main contributor for therapeutic effects can be of great importance, for example, for choosing of the route of administration.

Detection of other types of gut microbiota metabolites is necessary. At present, most studies that involve detection of gut microbiota metabolites mainly focus on SCFAs and the metabolites that are transformed from herbal medicines, for instance, ginsenosides. As aforementioned, there are a variety of bioactive metabolites produced by gut microbiota, such as secondary bile acids and biogenic amines. However, studies that focus on those metabolites in herbal medicine research area is scarcely seen. Some studies have hinted that herbal medicine could modulate those metabolites. For example, Don et al. found out that *Polygonum multiflorum* could perturbate the rat secondary bile acids including deoxycholic acid, hyodeoxycholic acid, ursodeoxycholic acid in bile and serum (Dong et al., 2015). The result indicates that, while studying the effect of *Polygonum multiflorum* on gut microbiota metabolites, detection of secondary bile acids is necessary. Therefore, except for SCFAs, detection of other types of gut microbiota metabolites is also necessary.

Integration of herbal medicine, gut microbiota, gut microbiota metabolites (including SCFAs), and phenotype is needed. Integration of these information could be helpful for understanding the mechanisms of herbal medicines at system level. Although a certain number of studies have investigated the influence of herbal medicines on gut microbiota and SCFAs, most of them just offer the information of the modulated gut microbiota species and SCFAs. In fact, gut microbiota, gut microbiota metabolites, and hosts is an interplaying system, therefore, integrating the information of them is crucial and necessary for comprehensive understanding the function of each building blocks of this system (Nicholson and Lindon, 2008; Knight et al., 2018). In our previous study, we used the integrative approach to study the mechanism of hydroxysafflor yellow A in reducing obesity and found out that *Bacteroidetes* and *Akkermansia* were negatively correlated with body weight (Liu et al., 2018). When the correlation of herbal medicine, gut microbiota, and phenotype is achieved, further steps such as fecal transplant of gut microbiota, gnotobiotic model, germ free animals are needed to strengthen the conclusion.

## Conclusions

As one type of metabolites of herbal medicines and food, SCFAs have been demonstrated to play important role in maintenance of homeostasis and recovery of diseases. In the past decade, most of the studies about herbal medicines and gut microbiota paid attention either to the effect of herbal medicines on gut microbiota composition or to the metabolites of gut microbiota. In recent years, the trend for studying herbal medicines has shifted toward detecting both gut microbiota composition and metabolites of herbal medicines. In this scenario, integration of information of herbal medicine, gut microbiota, gut microbiota metabolites, and phenotype is necessary. The integrative approach would be helpful for screening bioactive compounds and for understanding the mechanisms of herbal medicine at system level. In addition, since SCFAs, gut microbiota transformed herbal compounds, and untransformed prototype compounds might contribute to therapeutic effects, exploration of which of them contribute more to the therapeutic effects is needed. In this process, methods such as fecal transplant, gut microbiota culturing and animal models including gnotobiotic animal, germ free animal can be used. We believe that, with more and more attention paid to SCFAs and gut microbiota, our understanding and use of herbal medicines will be promoted.

## Author contributions

CP conceived and proposed the idea. WF designed, wrote and revised the manuscript. HA revised the manuscript.

### Conflict of interest statement

The authors declare that the research was conducted in the absence of any commercial or financial relationships that could be construed as a potential conflict of interest.
